# Influence of Culture Media Formulated with Agroindustrial Wastes on the Antimicrobial Activity of Lactic Acid Bacteria

**DOI:** 10.4014/jmb.2107.07030

**Published:** 2021-10-20

**Authors:** José R. Linares-Morales, Iván Salmerón-Ochoa, Blanca E. Rivera-Chavira, Néstor Gutiérrez-Méndez, Samuel B. Pérez-Vega, Guadalupe V. Nevárez-Moorillón

**Affiliations:** Facultad de Ciencias Químicas, Universidad Autónoma de Chihuahua. Circuito Universitario s/n, Campus II. C.P: 31125 Chihuahua, Chih. México

**Keywords:** LAB, food waste residues, by-product, fermentation, antimicrobial, pathogens

## Abstract

The discarding of wastes into the environment is a significant problem for many communities. Still, food waste can be used for lactic acid bacteria (LAB) growth. Here, we evaluated three growth media equivalent to de Mann Rogosa Sharpe (MRS), using apple bagasse, yeast waste, fish flour, forage oats, and cheese whey. Cell-free supernatants of eight LAB strains were tested for antimicrobial activity against nine indicator microorganisms. The supernatants were also evaluated for protein content, reducing sugars, pH, and lactic acid concentration. Cell-free supernatants from fish flour broth (FFB) LAB growth were the most effective. The strain *Leuconostoc mesenteroides* PIM5 presented the best activity in all media. *L. mesenteroides* CAL14 completely inhibited *L. monocytogenes* and strongly inhibited *Bacillus cereus* (91.1%). The strain *L. mesenteroides* PIM5 consumed more proteins (77.42%) and reducing sugars (56.08%) in FFB than in MRS broth (51.78% and 30.58%, respectively). Culture media formulated with agroindustrial wastes positively improved the antimicrobial activity of selected LAB, probably due to the production of antimicrobial peptides or bacteriocins.

## Introduction

It is well known that the manufacturing of many food products has led to the release of highly contaminated effluents into the environment, polluting soils and water bodies [1]. Fish production around the world for 2016 reached 171 million tons [2]; between 25 and 70% become wastes polluting the environment if they are not converted into valuable products [3]. Beer industry effluent is another waste of importance. It contains a considerable amount of yeast waste that reaches about 19 million kg per year worldwide [4]. Likewise, a large amount of whey is discarded during dairy products manufacturing, which is a contaminant with a high biological and chemical oxygen demand [5-7]. In 2011, approximately 200 million tons of whey were produced worldwide [8]. Between 2011-2012, close to 12.2 million metric tons of apples were used for juice production [9]; nearly 35%of that fruit was discarded as bagasse [10].

The problems caused by food waste can be reduced if they are included in bioconversion processes to contribute to circular economy strategies [5]. Using agroindustrial wastes to produce bioactive compounds through microbial fermentation as substitutes for culture media ingredients would result in a low-cost, environmentally friendly process [7]. Therefore, fermentation with lactic acid bacteria (LAB) can be an excellent way to reuse those agroindustrial wastes. Although LAB strains are fastidious microorganisms that demand culture-rich media, with a high content of nitrogen, minerals, and vitamins, they have a proteolytic system that allows them to obtain the essential amino acids needed for their growth, enabling them to grow well in protein-rich food [4, 7]. Peptone and yeast extract are expensive ingredients employed for the de Man, Rogosa, Sharpe culture medium (MRS) formulated for LAB's growth. Bustos *et al*. [11] and Sabo *et al*. [1] reported that only these ingredients account for almost 30% of MRS's total cost. However, these ingredients may be substituted by proteinaceous agroindustrial wastes, solving two of the most critical constraints for producing LAB metabolites for the food industry: ingredient availability and high cost [12, 13]. LAB strains have shown improved antimicrobial activity in substrates such as fish wastes [3, 14], whey [15] and were also able to produce bioactive peptides from whey [16] and lactic acid [17].

LAB satisfactorily produced biomass, lactic acid, and bacteriocin when grown in a culture medium formulated with fish protein hydrolysates [3]. Also, LAB produced a fermented protein hydrolysate with antimicrobial activity, probably due to the production of antimicrobial peptides when fermenting fish wastes [14]. LAB improved their antimicrobial activity against several pathogen microorganisms when whey was employed as a substrate; however, antimicrobial substances were not determined [15]. In addition, proteolytic LAB strains could release bioactive peptides from whey proteins [16] and produce biomass and improve the recovery of lactose, proteins, and minerals from clarified, heat-treated milk whey [17].

Moreover, the use of brewer's yeast wastes has enhanced LAB activities, possibly due to cellular lysis at the culmination of their active phase, releasing soluble metabolites to the medium [18, 19]. On the other hand, apple bagasse has been employed as a carbon source for lactic acid [18] and bacteriocin production [20] since it has a high content of soluble carbohydrates, which are crucial for biomass propagation [10, 20, 21]. Therefore, it is possible to consider using agroindustrial waste products to formulate alternative growth media for LAB growth. In a previous report [22], LAB isolated from vegetable sources were screened according to their proteolytic and antimicrobial activities by microtitration in MRS broth. Eight LAB strains (3 *Enterococcus mundtii*, 2 *Enterococcus faecium*, and 3 *Leuconostoc mesenteroides*) demonstrated the best antimicrobial activities against pathogenic microorganisms. Following the characterization of selected LAB strains, our objective in this research was to evaluate their antimicrobial activity compared to MRS broth when they were grown in three different culture media formulated with agroindustrial wastes as protein source.

## Materials and Methods

### Microorganisms

LAB, indicator microorganisms, and incubation conditions are listed in Table 1.

LAB isolated from vegetable sources were reported previously and selected based on their protease and antimicrobial activity against several indicator microorganisms [22].

### Characterization of Agro-Industrial Wastes

The agroindustrial wastes employed were apple bagasse (AB) from Grupo La Norteñita (México), and yeast water waste (YW) from Tijarare Cervecería Artesanal (Mexico). Previously, YW was mixed with sterile water and allowed to decant for 30 min. The darkest phase was separated, and the rest was taken as yeast water. Commercial fish flour (FF) was purchased at a local supermarket; cheese whey (CW) was donated by the Cheese Factory at the Animal Science Department, Universidad Autónoma de Chihuahua, and foraged oats, FO, (*Avena sativa* L. var. Karma) was obtained at Semillas Pura Sangre (Mexico). All agroindustrial wastes were analyzed to determine protein content and total nitrogen by the Kjeldahl method [23] and reducing sugars as glucose using the 3,5 dinitrosalicylic acid method [24].

### Culture Media Formulation

Once the agroindustrial wastes were characterized, three culture media with similar proximal composition to MRS broth were formulated. Agroindustrial wastes were used as substitute ingredients in the equivalent amounts of carbon and nitrogen supplied in the MRS broth (Difco, USA), as reported by the company and indicated by the approximate amount of C and N supplied by peptone, yeast extract, and meat extract. To calculate the C/N ratio, the C and N proportion provided by each component in the media formula was stoichiometrically calculated. Concentrations of Tween 80 and inorganic salts were maintained in equivalent amounts as in MRS broth. A culture medium base was prepared with AB and YW (substituting glucose and yeast extract, respectively) Tween 80 and inorganic salts. Later, three culture broths were prepared, each with a different protein source substituting for peptone and meat extract. The three media were fish flour broth (FFB), cheese whey broth (CWB), and forage oats broth (FOB), formulated with commercial fish flour (FF), whey (CW) from the cheese industry, and forage oats (FO) as the protein sources respectively.

### Fermentation of Culture Broths Formulated with Agroindustrial Wastes

To determine the influence of the formulated media on the antimicrobial activity of the selected LAB isolates, MRS broth and the different media were inoculated with 5 ml of an overnight growth of the LAB isolates tested. The broths were incubated at 26°C for 48 h. Later, the supernatants were centrifuged at 3,800 ×*g* for 25 min (5810R, Eppendorf, Germany) and pasteurized (75°C, 3 min) to obtain the cell-free supernatants (CFS). LAB growth and preparation of the CFS were previously reported, while the methodology for evaluating the antimicrobial activity of the CFS of the LAB isolates also followed a previously reported method [22]. All experiments were carried out in triplicate.

To determine the influence of the formulated media on the antimicrobial activity of the selected LAB isolates, MRS broth and the different media were inoculated with the eight LAB isolates tested. A sample loop of each LAB strain was inoculated in tubes with 10 ml of MRS broth. Later, 5 ml of the overnight growth of each LAB strain was inoculated into 125 ml Erlenmeyer flasks containing 45 ml of each formulated media and MRS broth. The flasks were stationarily incubated at 26°C for 48 h. Later, the supernatants were centrifuged at 3,800 ×*g* for 25 min (5810R, Eppendorf, Germany) and pasteurized (75°C, 3 min) to obtain the cell-free supernatants (CFS). All experiments were carried out in triplicate.

### Antimicrobial Activity of the Cell-Free Supernatants

Each CFS's antimicrobial activity was evaluated as the percentage of inhibition of the growth of indicator microorganisms following the microtiter method described by Linares-Morales *et al*. [22]. Briefly, 100 μl of Brain Heart Infusion (BHI) broth for bacteria (Bioxon, México) and YM broth (yeast extract 3 g/l, malt extract 3 g/l, dextrose 10 g/l, peptone 5 g/l) for yeast and molds were transferred to a 96-well microplate. To avoid the inhibitory effect of lactic acid produced by LAB, broth media were supplemented with K_2_HPO_4_ (0.1 M). To each well, 30 μl of indicator microorganism suspensions (Table 1) was added, with a concentration of 1.5 × 10^8^ cells/ml for bacteria and yeast (0.5 McFarland standard) and 10^3^-10^4^ mold spores (adjusted based on New Bauer chamber counts). Furthermore, 50 μl triplicates of CFS previously was centrifuged at 3,800 ×*g* for 25 min (5810R, Eppendorf, Germany) and pasteurized (75°C, 3 min) were added. Lastly, 150 μl of the supplemented media and 30 μl of the microbial suspensions were incorporated as negative controls. The optical density at 595 nm (OD_0_) was determined with a microplate reader (Bio-Rad 550, Japan) as the initial lecture. Microplates were incubated following the conditions stated in Table 2 for each microorganism. When incubation finished, optical density (OD_f_) was obtained again, and the antimicrobial activity (AA) was calculated employing the following equation:

AA = 100 - ((DDOs/DDOc) × 100).

DDOs are the average difference between the samples' initial (OD0) and final (ODf) optical density. DDOc is the average of the difference (OD_0_ and OD_f_) of the optical density of the negative control. AA was classified as follows: higher than 50% was considered strong inhibition, between 15 and 49.99% moderate inhibition, and lower than 14.99% was considered no inhibition. Indicator microorganisms were incubated in sterilized culture media formulated with agroindustrial wastes to determine whether the formulated media presented an antimicrobial effect.

### Chemical Analysis of Culture Broths and Cell-Free Supernatants from LAB Growth

Consumption of proteins and sugars, pH, and lactic acid production by the LAB strain with the best antimicrobial activity were determined. Thus, protein content by Bradford method [25], reducing sugars as fructose by the 3,5 dinitrosalicylic acid method [24], total titratable acidity (TTA) by the AOAC Official Method 942.15 [23], expressed as the amount in grams of lactic acid, and pH by the AOAC Official Method 981.12 [23], were determined for the CFS and the sterile broth and compared to the MRS broth.

### Statistical Analysis

The effect of the culture media on the LAB's antimicrobial activity was subjected to an analysis of variance (ANOVA) followed by a Tukey simultaneous test for means differences. Statistical analyses were done using the Minitab 18 software (Minitab Inc., USA), with a significance level of *p* < 0.05.

## Results

### Culture Media Formulation

Table 2 presents the reducing sugars, nitrogen, and proteins of the five agroindustrial wastes used to formulate the culture media for LAB growth. AB presented the highest content of reducing sugars (62.41%), followed by YW (9.84%) and FF (6.69%). YW's nitrogen content (4.52%) and FF (3.99%) were higher than that of CW. Three culture media were formulated, considering the composition of the agroindustrial waste used, with equivalent amounts of the carbon provided by glucose and nitrogen provided by yeast extract, peptone, and meat extract present in the MRS broth; the composition of salts and the concentration of Tween 80 were maintained as in the MRS formulation (Table 3). The carbon/nitrogen ratios (C/N) of the three formulated culture media, FFB (3.55), FOB (3.43), and CWB (3.22), were lower than the C/N ratio of the MRS broth (4.06).

### Antimicrobial Activity

**Fig. 1:**
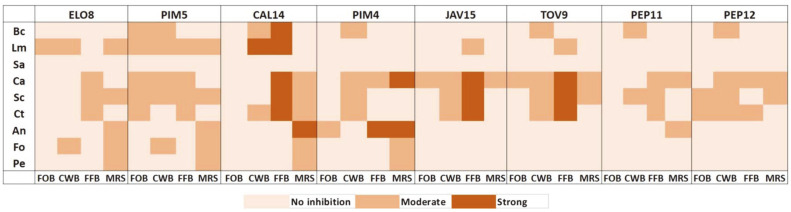
Comparison of the antimicrobial activity of LAB-CFS against the indicator microorganisms per culture media. Strain of lactic acid bacteria: *Enterococcus mundtii* ELO8, *Leuconostoc mesenteroides* PIM5, *Leuconostoc mesenteroides* CAL14, *Enterococcus faecium* PIM4, *Enterococcus mundtii* JAV15, *Enterococcus mundtii* TOV9, *Enterococcus faecium* PEP11, *Leuconostoc mesenteroides* PEP12. Classification of antimicrobial activity: > 50% = strong inhibition; 15-50% = moderate inhibition; <14.99% = no inhibition. Culture media: FOB: Forage oats broth, CWB: Cheese whey broth, FFB: Flour fish broth. Indicator microorganisms: Bc: *B. cereus*, Lm: *L. monocytogenes*, Sa: *S. aureus*, Ca: *C. albicans*, Sc: *S. cerevisiae*, Ct: *C. tropicalis*, An: *A. niger*, Fo: *F. oxysporum*, Pe: *P. expansum*.

Fig. 1 shows the AA of LAB-CFS over indicator microorganisms compared among the formulated culture media and the MRS broth.

The FOB was the medium where the CFS from LAB showed less AA. Only *L. mesenteroides* PIM5 was able to inhibit the growth of more than two indicator microorganisms when cultured in FOB. The best antimicrobial activity was observed in the FFB where three LAB-CFS achieved strong inhibitions and three more achieved moderate inhibitions on all the yeasts evaluated. Moreover, four CFS of the LAB strains (*L. mesenteroides* PIM5 and CAL14, *E. mundtii* JAV15 and TOV9) could inhibit the growth of at least four indicator microorganisms when these LAB were grown in FFB. On the other hand, the second-best AA was observed in the MRS broth. Although four LAB-CFS achieved moderate inhibitions against at least four indicators, only three exerted strong inhibitions, and only one LAB-CFS moderately inhibited all the yeasts. Regarding the LAB strains, *L. mesenteroides* CAL14 completely inactivated or significantly reduced the growth of *B. cereus*, *L. monocytogenes*, and the yeasts assayed. However, *L. mesenteroides* PIM5 displayed the best antimicrobial profile since it could inhibit at least four indicator microorganisms in each culture media employed.

The antimicrobial activity of LAB-CFS, when grown in media formulated with agroindustrial wastes against indicator microorganisms, is incorporated in Table 1 of Supplementary Information (TS1). The CFS's antimicrobial activity from LAB was significantly affected (*p* < 0.05) by the culture media and the LAB strain.

The CFS of *L. mesenteroides* PIM5 grown in FOB was the only CFS able to moderately inhibit the growth (*p* < 0.05) of more than two indicator microorganisms (*B. cereus* 35.5% ± 4.5, *L. monocytogenes* 46.7% ± 8.6, *C. albicans* 39% ± 5.5, *S. cerevisiae* 45.2 % ± 14.9 and *C. tropicalis* 49.2% ± 15.1). Regarding the antimicrobial activities of the CFS from LAB grown in the CWB, *L. mesenteroides* PIM5 CFS moderately inhibited the growth (*p* < 0.05) of five indicator microorganisms (*B. cereus* 39.5% ± 6.6, *L. monocytogenes* 45.9 % ± 4.3, *C. albicans* 34.4 % ± 7.7, *S. cerevisiae* 43.1 % ± 11.5 and *F. oxysporum* 23.3 % ± 3.7). Moreover, the CFS of the strains *E. faecium* PIM4, *E. mundtii* TOV9 and *L. mesenteroides* PEP12 presented moderate inhibition (*p* < 0.05) against *B. cereus* and the yeasts evaluated. About the FFB, the CFS of the strain *L. mesenteroides* CAL14 completely inhibited the growth (*p* < 0.05) of *L. monocytogenes* (114.2 % ± 53.5) and strongly inhibited the growth of *B. cereus* (91.1 %± 20.6), *C. albicans* (74.6 % ± 2.6), *S. cerevisiae* (80.4 % ± 6.7) and *C. tropicalis* (62.8 % ± 3.4). Furthermore, the CFS of *L. mesenteroides* PIM5 moderately inhibited the growth (*p* < 0.05) of *L. monocytogenes* (27.9 % ± 21), *C. albicans* (39 % ± 5.5), *S. cerevisiae* (45.2 % ± 14.9), and *C. tropicalis* (49.2 % ± 15.1). When the LAB employed were cultured in MRS broth, the strain *L. mesenteroides* CAL14 reduced the growth (*p* < 0.05) of yeast (*C. albicans* 47.3 % ±7, *S. cerevisiae* 16.4 % ± 6.1, *C. tropicalis* 20.7% ± 10.9) and molds (*A. niger* 58.3 % ± 5, *F. oxysporum* 22.2 % ± 16.7, *P. expansum* 33.4 % ± 17.9). Likewise, *E. mundtii* ELO8 and *L. mesenteroides* PIM5 were able to inhibit moderately the growth (*p* < 0.05) of *L. monocytogenes*, *S. cerevisiae*, and molds.

Antimicrobial activities of sterile culture media formulated with agroindustrial wastes and MRS broth against indicator microorganisms (Table 4) demonstrated that the MRS broth and none of the broths formulated with agroindustrial wastes exerted antimicrobial activity except for the CWB, which inhibited the growth of *C. albicans* (22.6 % ± 7.2) and *S. cerevisiae* (16.9 % ± 9.4).

### Chemical Analysis

The chemical analysis of the CFS of *L. mesenteroides* PIM5 grown in FFB and MRS is shown in Table 5. The values for pH and lactic acid production were similar in both CFS. However, protein consumption and the concentration of reducing sugars were higher in FFB than in MRS broth. Less than a quarter of the initial proteins present in FFB remained in the CFS, whereas less than half of the MRS broth proteins remained in its CFS. Regarding the uptake of the reducing sugars, more than half was consumed in FFB, while in MRS, only over a third of the reducing sugar content was consumed. The results demonstrate the differences in components consumption in both formulations.

## Discussion

Culture media equivalent to MRS broth were formulated substituting carbon, nitrogen, and peptone sources with agroindustrial waste to assess the influence of the components on the antimicrobial activity of LAB CFS. A base culture medium was formulated, adding apple bagasse (AB) instead of glucose as a carbon source and yeast water waste (YW) instead of yeast extract as a nitrogen, amino acid, and vitamin source. Inorganic salts were added in the same concentration as in the MRS broth original formulation. Three culture media were formulated, adding different protein sources (fish flour, forage oats, and whey) to substitute for peptone and meat extract. Each source was added in equivalent quantities as for the nitrogen provided by peptone and meat extract. All the agroindustrial wastes employed have already been evaluated as substrates for lactic acid fermentation. LAB successfully fermented apple bagasse for lactic acid production [18], brewer's yeast wastes for protease production [4], forage oats for silage [26], hydrolyzed fish viscera as a growth substrate [27], and whey for bacteriocin production [1].

AB was revealed to be a good carbon source for lactic acid fermentation; according to the chemical analysis, it contains an elevated concentration of simple sugars, as determined by the reducing sugar concentration. The percentage of available sugars will depend on the fruit variety and the method employed for juice preparation [28]. However, the content of reducing sugars in AB (62.41%) was close (68%) to that reported by Gullón *et al*. [18]. Based on this concentration, the amount of AB added to the formulated medium was equal to that supplied by the glucose incorporated into the MRS broth (8 g C /L). Fructose is the major component of the sugars contained in AB [18]. When this sugar is present in a culture medium, *L. mesenteroides* can employ a portion as an electron acceptor to reduce fructose to mannitol, thus acquiring energy. Another portion is transformed to lactic and acetic acid and even to small amounts of ethanol [20].

Moreover, a strain of *Lactobacillus amylovorus* showed the same growth rate with a mixture of glucose and fructose than when glucose was used as the only carbon source [20]. Besides, LAB can transform oligosaccharides into single sugars that can be efficiently absorbed. For instance, LAB strains can obtain fructose from the fructans present in apple bagasse [29]. Therefore, a sugar mix ingredient like AB would sustain LAB growth. Although proteins are also a constituent of AB, their content is low [28], as confirmed in this report.

The protein content of YW from beer production was lower than previously reported by dos Santos Mathias *et al*. [19]. Yeast extract is a good source of nitrogen and carbon; nevertheless, its percentages would change according to their physiological state and growth phase [28]. Still, the amount of nitrogen and carbon provided by YW was calculated to add an amount equivalent to that provided by the commercial yeast extract used in the MRS broth. Yeast waste is rich in soluble proteins due to yeast's autolysis at the end of fermentation and sterilization [19]. It is also a good source of amino acids (lysine, leucine, isoleucine, and tryptophan), B vitamins, purine and pyrimidine bases, and minerals that influence the growth of LAB during lactic acid fermentation [7, 11, 19]. Yeast waste from the brewing industry has already been proved to cause an increase in LAB biomass, a reduction in fermentation time [28], and complete depletion of glucose and fructose [18].

Furthermore, yeast water waste from beer production has a low C/N ratio due to its high carbon content, which enhances biomass production; therefore, it has high potential for being used as an ingredient in fermentation culture media [19]. FO nitrogen content was similar to previous reports [26, 30]. FO is a suitable substrate for lactic acid fermentation since there are several LAB isolated from this substrate. It contains a good proportion of proteins, carbohydrates, and minerals to support LAB growth [26, 30]. Similarly, whey has a significant amount of proteins, lipids, vitamins, and minerals, in addition to lactose [5] available for LAB growth. However, the nitrogen content of CW was lower than the nitrogen provided by peptone and meat extract in the MRS broth. Thus, the broth was prepared by adding all the ingredients to the CW without adding extra water. The total protein of FF (24.91 g/l) was lower than that reported by Vázquez *et al*. [3] for fish wastes (31.3 to 43 g/l). Nonetheless, the nitrogen content provided by FF and added to the FFB was calculated to add an equal amount to that supplied by peptone and meat extract in MRS formulation.

Although the nitrogen provided by agroindustrial wastes as protein sources was equal to the amount provided by the MRS broth, the C/N ratio was lower in the formulated media than in the MRS broth due to a lower carbon content in these agroindustrial sources than that supplied by peptone and meat extract in the MRS broth. The C/N ratio is a key characteristic for culture media to accomplish sufficient growth and lactic acid fermentation. Carbon sources are used in LAB cells for energy production, while nitrogen, vitamins, and minerals produce biomass, lactic acid, and cell maintenance [7, 13]. A culture medium with a high C/N ratio would help produce bioactive metabolites from proteolytic LAB [19].

Agroindustrial wastes employed as protein sources favored LAB's antimicrobial activity, but it varies according to the protein source supplied and the LAB strain. The method used for the evaluation of antimicrobial activity was selected based on our previous results. The buffered broth was able to neutralize the lactic acid present in the CFS. The method can detect a reduction in the microbial growth even when the inhibition is not complete; therefore, it was possible to assess differences that could not be observed with other methods such as the spot-on-the-lawn or the determination of minimal inhibitory or bactericidal concentrations. The method also allowed us to test the CFS against more indicator microorganisms, including fungi, yeast, and bacteria [22].

The antimicrobial activity might be due to the production of antimicrobial peptides released from the FF by proteases of LAB since the media's pH was buffered to avoid the action of acids produced by LAB [14], and all these LAB were shown to be proteolytic strains [22]. These results are similar to Ruthu *et al*. [14], who also reported the inhibition of *B. cereus* and *L. monocytogenes* by a protein hydrolysate obtained from fish wastes fermented by LAB. Antimicrobial peptides can be obtained at a lower cost by enzymatic hydrolysis by employing LAB proteolytic activity [16]. Liu *et al*. [31] report that the LAB proteolytic system comprises cell-wall bound proteinase hydrolyzing protein into oligopeptides, peptide transporters in charge of transferring the peptides to the cell, and intracellular peptidases in charge of degrading the transferred peptides into smaller peptides and amino acids. Furthermore, protein breakdown is favored by lactic acid production since it disturbs protein structure, enhancing protease activity [32]. Besides the production of antimicrobial peptides by LAB, bacteriocins production is another possibility. A strain of *Lactobacillus plantarum* produced bacteriocins from a medium supplemented with whey [1]. *Pediococcus acidilactici* produced pediocin from fish wastes hydrolysates [3]. Strains of *L. mesenteroides* [33, 34], *Enterococcus mundtii* and *Enterococcus faecium* [35] have also been reported as bacteriocin producers.

Whey components are mainly lactose and whey proteins; they also consist of amino acids, vitamins, and salts of calcium, potassium, sodium, and magnesium [6, 17]. Besides lactose fermentation capacity, LAB proteolytic systems can release antimicrobial peptides from proteins provided by whey [16, 36] like casein, β-lactoglobulin, α-lactalbumin, and serum albumin. Therefore, whey is suitable for LAB growth and production of antimicrobial peptides [1]. For instance, Theolier *et al*. [36] were able to identify fractions of β-lactoglobulin and α-lactalbumin with antimicrobial activity after hydrolyzing whey. However, lactoferrin, lactoperoxidase, and lysozyme present in whey have been shown to exert antimicrobial activities [8, 36]. This observation was confirmed when *C. albicans* and *S. cerevisiae* were incubated with sterilized CWB, and their growth was partially inhibited. Therefore, the partial inhibition of *C. albicans* and *S. cerevisiae* when grown in sterilized CWB can result from antimicrobial whey proteins. Another possibility is that proteins are partially hydrolyzed during cheese production [36], which opens the probability that antimicrobial peptides present in the whey could have exerted such inhibition of yeasts. Similarly, peptides with antimicrobial activity against yeasts could be released during sterilization of the CWB, remaining in the sterilized medium.

Phenolic compounds present in apple bagasse do not affect *Leuconostoc* spp. since they can survive in plants due to their tolerance to high amounts of phenolic compounds which they can metabolize, producing metabolites with less antimicrobial activity [29].

In the present research, lactic acid production in the FFB and MRS was similar, as previously reported for a fructose-containing medium [12]. However, a better nutrient uptake was determined for *L. mesenteroides* when fructose was present in the medium [33], which explains the preferential consumption of protein and reducing sugars in FFB compared to MRS broth. The uptake of nutrients in the culture media formulated with agroindustrial wastes would produce an expended medium with less organic load reducing the biological oxygen demand [17].

Culture media formulated with agroindustrial wastes proved to positively influence the antimicrobial activity of LAB isolated from fresh vegetable sources. The fish flour broth was the medium that most encouraged the antimicrobial activity of most of the LAB strains evaluated. However, antimicrobial activity is a strain-dependent feature, and therefore, production and characterization of antimicrobial peptides by *Leuconostoc mesenteroides* PIM5 must be further investigated since this strain presented the best antimicrobial profiles against the indicator microorganisms assayed in the three media formulated.

## Supplemental Materials

Supplementary data for this paper are available on-line only at http://jmb.or.kr.

## Figures and Tables

**Table 1:** Lactic acid bacteria and indicator microorganisms for the antimicrobial activity test.

Group	Microorganism	Accession number/ATCC	Conditions
LAB	*Enterococcus mundtii* ELO8	MN636717	26°C, 24 h Microaerophilic
	*Leuconostoc mesenteroides* PIM5	MN636704	
	*Leuconostoc mesenteroides* CAL14	MN636705	
	*Enterococcus faecium* PIM4	MN636718	
	*Enterococcus mundtii* JAV	MN636719	
	*Enterococcus mundtii* TOV9	MN636706	
	*Enterococcus faecium* PEP11	MN636707	
	*Leuconostoc mesenteroides* PEP12	MN636720	
	*Bacillus cereus*	11778	
Gram +	*Listeria monocytogenes*	19114	37°C, 24 h
	*Staphylococcus aureus*	25923	Aerobiosis
	*Aspergillus niger*	BUAP* collection	26°C, 48 h
Molds	*Penicillium expansum*	UDLAP** collection	Aerobiosis
	*Fusarium oxysporum*	UDLAP** collection	
	*Candida albicans*	10231	26°C, 24 h
Yeasts	*Saccharomyces cerevisiae*	9763	Aerobiosis
	*Candida tropicalis*	1369	

*BUAP: Benemérita Universidad Autónoma de Puebla. **UDLAP: Universidad de las Américas Puebla.

**Table 2:** Characterization in percentages of agro industrial wastes employed to formulate three culture media similar to MRS broth.

Variable	AB	YW	FO	CW	FF
Red sugars	62.41 ± 0.06	9.84 ± 0.00	2.49 ± 0.01	0.01 ± 0.00	6.69 ± 0.02
Nitrogen	0.66 ± 0.21	4.52 ± 0.33	1.7 ± 0.03	0.16 ± 0.01	3.99 ± 0.11
Protein	4.79± 0.9	28.25 ± 2.07	10.63 ± 0.22	1.02 ± 0.32	24.91 ± 0.70

AB: Apple bagasse, YW: Yeast waste, FO: Forage oats CW: Cheese whey FF: Fish flour.

**Table 3:** Content in g/l of MRS broth compared to three culture media formulated with agro industrial wastes as protein source.

Ingredient	Medium				Nutrient	

	MRS	FOB	CWB	FFB	Carbon	Nitrogen
Yeast extract	5				~0.5	~0.5
Glucose	20				8	0
Peptone	10				~3	~1.4
Meat extract	10				~3	~1
Tween 80	1	1	1	1	0.59	0
Ammonium citrate	2	2	2	2	0.64	0.99
Sodium acetate	5	5	5	5	0.88	0
Magnesium sulphate	0.2	0.2	0.2	0.2	0	0
Manganese sulphate	0.05	0.05	0.05	0.05	0	0
Dipotassium phosphate	2	2	2	2	0	0
Yeast water		11 ml	11 ml	11 ml	0.5	0.5
Apple bagasse		32.01	32.01	32.01	8	0.2
Forage oats		141			3.51	2.4
Cheese whey			1000 mL		0.1	1.6
Fish flour				60	4	2.4
C/N	4.06	3.43	3.22	3.55		

FFB: Flour fish broth, FOB: Forage oats broth, CWB: Cheese whey broth.

**Table 4:** Antimicrobial activities of sterile culture media formulated with agro industrial wastes and MRS broth against indicator microorganisms.

Indicator/Media	FFB	FOB	CWB	MRS
*B. cereus*	-43.67 ± 18.3	-81.7 ± 22.2	-28.9 ± 22.8	-85.3± 3.4
*L. monocytogenes*	-51.04 ± 26.1	-93 ± 30.4	-30.1 ± 25.6	-102.5 ± 30.1
*S. aureus*	-220.24 ±51.9	-209.2 ± 88.8	-192.3 ± 53	-377.1 ± 16.2
*C. albicans*	-1.8 ± 9.7	-1.1 ± 12.5	22.6 ± 7.2	-43.9 ± 7.7
*S cerevisiae*	-8.6 ± 7.2	-4.1 ± 9.3	16.9 ± 9.4	-14.1 ± 5.4
*C tropicalis*	-32.4 ± 17.1	-30.8 ± 13.4	-11.7 ± 5.5	-46.8 ± 2.8
*A. niger*	-19.7 ± 16.6	-41.9 ± 5.3	-23.2 ± 3.3	5.3 ± 15
*F. oxysporum*	-32.2 ± 9.9	-26.6 ± 6.4	-33.6 ± 7.3	-11 ± 5.5
*P. expansum*	-129.5 ± 19.5	-146.9 ± 7.3	-96.2 ± 10.7	-33.9 ± 2.9

FFB: Flour fish broth, FOB: Forage oats broth, CWB: Cheese whey broth.

**Table 5:** Performance of the strain *L. mesenteroides* PIM5 in FFB and MRS broth.

Variable	FFB		MRS	

	Sterile broth	CFS	Sterile broth	CFS
pH	6.2 ± 0.00	4.35 ± 0.03	6.52 ± 0.00	4.38 ± 0.00
Titratable acidity g/100 ml	0.09 ± 0.00*	0.80 ± 0.01**	0.22 ± 0.01***	0.79 ± 0.02**
Proteins g/l	2.17 ± 0.00	0.49 ± 0.06	0.56 ± 0.00	0.27 ± 0.01
Reducing sugars g/l	19.01 ± 0.18	8.35 ± 0.19	18.90 ± 1.09	13.12 ± 0.68

*g of malic acid. **g of lactic acid, ***g of acetic acid
