# Supplementary material for: Influence of Culture Media Formulated with Agroindustrial Wastes on the Antimicrobial Activity of Lactic Acid Bacteria
Source: J Microbiol Biotechnol. 2021 Oct 20;32(1):64–71. doi: 10.4014/jmb.2107.07030 (PMC9628826; doi:10.4014/jmb.2107.07030)
Supplement: Supplementary file 1 [file jmb-32-1-64-supple.pdf]

J. Microbiol. Biotechnol.
https://doi.org/10.4014/jmb.2107.07030

Supplementary Table

Table 4. Antimicrobial activity of the cell-free supernatant from lactic acid bacteria grown in media formulated with agroindustrial wastes.

	ELO8	PIM5	CAL14	PIM4	JAV15	TOV9	PEP11	PEP12
					FOB			
**Bc**	-8.2±10^{abc}	35.5±4.5^{ab}	-14.7±20.2^{abc}	-5±9.4^{abc}	-84.5±13.9^{cd}	-84.1± 18.2^{cd}	-27.1±13.4^{bcd}	-16.3±18.9^{abc}
**Lm**	15±2^{ab}	46.7±8.6^{ab}	-27.7±19.4^{abc}	-154.1±18.8^{cd}	-3.9±31.1^{ab}	4.8±27.6^{ab}	-132±23.2^{bc}	-21.5±15.8^{abc}
**Sa**	-549±78.2^{d}	-255±70.9^{cd}	-81.3±22.4^{abc}	-116.3±44.2^{abc}	-30.6±24.6^{ab}	-9.7±24.2^{ab}	-215.6±10.2^{bc}	-66.3±9^{abc}
**Ca**	5.2±1.9^{a}	39±5.5^{a}	9.17±5.5^{a}	-0.6±8.7^{a}	24.9±13.8^{a}	21.7±17.4^{a}	3.1±18.5^{a}	10.6±20.2^{a}
**Sc**	-2.2±5.9^{a}	45.2±14.9^{a}	9.64±2.5^{a}	-1.5±22.3^{a}	13.3±14.6^{a}	14.2±17.4^{a}	-1.4±14.2^{a}	19.3±20^{a}
**Ct**	-72.3±15.4^{ade}	49.2±15.1^{cde}	6.58±13.9^{bcd}	-3.6±13.1^{abcde}	10.3±5.8^{bcd}	10.6±9.8^{bcd}	-24.9±2.2 ^{abcde}	18.6±5.6^{bcd}
**An**	-1.5±5.6^{ab}	-41.6±15.5^{abc}	0.2±1.1^{ab}	29.2±25.2^{ab}	-111.1±23.3^{cde}	-138.7±12.5^{de}	-68.7±4.9^{bcd}	-70.6±4.5^{bcd}
**Fo**	13.4±11.5^{abc}	6.1±6.6^{abc}	-4.4±6.1^{abc}	-3.4±8.15^{abc}	-103.6±37.8^{cde}	-94.1±43.7^{cde}	-30,3±38.3^{abc}	-77.8±14.7^{bcde}
**Pe**	7.4±13.1^{a}	-14.5±5.1a	-24.7±27.1^{a}	-27.4±10.3^{a}	-215.1±66.3^{b}	-218.5±47.7^{b}	-153.5±50.6^{b}	-209.2±31.4^{b}
					CWB			
**Bc**	5±8.5^{a}	39.5±6.6^{a}	47.9±27.4^{a}	56.2±22.7^{a}	7±39.9^{a}	19.3±47.7^{a}	15.5±17.5^{a}	25.5±1.7^{a}
**Lm**	20.7±12.1^{abc}	45.9±4.3^{abc}	56.5±34.9^{a}	-54±58.6^{abc}	-1.1±4.5^{abc}	-44.3±99.6^{abc}	-69.9±40.3^{c}	13.9±8.1^{abc}
**Sa**	-572.6±112.2^{b}	-459.1±28^{b}	-57.5±38.4^{a}	-64.8±12.2 ^{a}	-54.8±16^{a}	-76.3±27.9^{a}	-166.6±18^{a}	-73±14^{a}
**Ca**	4.1±1.4^{abc}	34.4±7.7^{ab}	6.8±20^{abc}	40.5±4.6^{ab}	31.1±7.2^{abc}	35.4±7.7^{ab}	-9.2±21.7^{ac}	39.5±5.7^{abc}
**Sc**	12.3±2^{a}	43.1±11.5^{a}	14.9±9.7^{a}	15.8±8.2^{a}	30.7±10.7^{a}	32.7±10.8^{a}	19.5±15.3^{a}	47.2±6^{a}

**Ct**	-31.5 ± 29.9^{cde}	13.6 ± 3.7^{abcde}	48.7± 1.1^{abc}	34.7±16.5^{abcd}	23.3±3.1^{abcd}	26.7 ± 6.2^{abcd}	-8.2 ± 11^{bcde}	32.5±3.6^{abcd}
**An**	-128.3±2.9^{cd}	-2.3±12.6^{ab}	-202.8±44.6^{e}	-13.8±14.8^{ab}	-79.7±31.7^{bcd}	-117.7±29.9^{cd}	-92.2±7.5^{bcd}	-50.9±2.8^{abc}
**Fo**	16±7.2^{a}	23.3±3.4^{a}	-3.6±4.9^{a}	-2.8±4.6^{a}	-129.6±19.7^{b}	-114.5±30.1^{b}	-9±19.3^{a}	-24.2±17.4a
**Pe**	-1.1±3.3^{a}	6.3±11.9^{a}	-33.7±2.7^{a}	-29.9±7.3^{a}	-155.5±66^{b}	-171.1±50.6^{b}	-172±7.2^{b}	-117.3±3.2b
				**FFB**				
**Bc**	0.0±7.8^{ab}	-20.8±17.9^{abc}	91.1±20.6^{d}	11.7±23.7^{ab}	-62.8±7.7^{bc}	-60.8±8.8^{bc}	-22.8±11.5^{abc}	-15.9±9.5^{bcd}
**Lm**	0.8±8.3^{abc}	27.9±21^{abc}	114.2±53.5^{ab}	-127.3±39.6^{cd}	18.7±49.1^{abc}	17.8±38^{abc}	-63.4±99.8^{bc}	4.6±72.7^{abc}
**Sa**	-380.2±42.6^{c}	-255±70.9^{bc}	-11.8±22.4^{ab}	-127.3±33.8^{ab}	5.4±28^{ab}	-3.2±14.2^{ab}	-121±112^{ab}	-63±121.7^{ab}
**Ca**	26±5.3^{bcde}	39±5.5^{abcde}	74.6±2.6^{abc}	25.1±12^{bcde}	75.5±3.6^{abc}	67.1±2.8^{abcd}	20±42.4^{cde}	55.2±32.8^{abcde}
**Sc**	47.5±25.7^{ab}	45.2±14.9^{ab}	80.4±6.7^{ab}	-11.5±7.7^{a}	75.5±7.5^{ab}	73.7±5.9^{ab}	31±48.9^{abc}	-63±121.7^{ab}
**Ct**	37.3±17.4^{abcde}	49.2±15.1^{abc}	62.8±3.4^{abcd}	-3.2±5.7^{cde}	78.3±8^{abc}	72.8±5.3^{abc}	21.1±35.9^{bcde}	41.1±45.4^{abcde}
**An**	-45.9±9.2^{bc}	-41.6±15.5^{bc}	-7.5±2.8^{bc}	50.2±13.5^{a}	-107.3±6.7^{de}	-99.6±4.3^{cde}	-41±14.6^{bc}	-47.1±3.8^{bcd}
**Fo**	-0.3±1.5^{a}	6.1±6.6^{a}	11.8±3.8^{a}	-2.2±4.7^{a}	-130.2±40.4^{b}	-126±9.6^{b}	-42.2±46.7^{a}	-48.9±24.4^{a}
**Pe**	-16.9±4.6^{a}	-14.5±5.1^{a}	-18.6±5.9^{a}	-27.8±12.8^{a}	-223.3±12.1^{b}	-217.1±16.4^{b}	-154.1±56.4^{b}	-187.1±22.3^{b}
				**MRS**				
**Bc***	7.6±22.8^{a}	-1.6±12.6^{a}	-4.5±8.2^{a}	-16.8±2.8^{a}	-42.7±34.1^{a}	-46.9±10.2^{a}	-1.8±15.7^{a}	-19.2±9.6^{a}
**Lm†**	22.3±9.5^{abc}	40±11^{ab}	-18.4±13.2^{abc}	-41.5±17.3^{abc}	-40.8±29.2^{abc}	-69.6±25.7^{abc}	-93.7±6.3^{bc}	-12.5±7.6^{abc}
**Sa‡**	-504.7±33^{f}	-235.9±90.8^{bcde}	-9.1±22.2^{abc}	-47.8±13.6^{abc}	-73.4±30.9^{abc}	-82.6±23.7^{abc}	-248.3±7.7^{cde}	-104.3±52.5^{abc}
**Ca§**	-1±1^{bc}	10.1±10.4^{abc}	47.3±7^{ab}	55.9±4.7^{ab}	28.5±26.6^{abc}	29.6±10.2^{abc}	15.9±18.6^{abc}	37.4±10.6^{abc}
**Sc¶**	21±8.1^{a}	20.3±8.1^{a}	16.4±6.1^{a}	5.4±2.5^{a}	11±16.3^{a}	19±9^{a}	5.2±9.5^{a}	31.8±8.7^{a}
**Ct****	-61.6±5.1^{cde}	-37.1±7.7^{bcde}	20.7±10.9^{abc}	12.1±3.8^{abcd}	5.6±6.7^{abcd}	7.9±4^{abcd}	-27.7±2.9^{abcde}	9.4±2.3^{abcd}

**An††**	39.2±30.4^{a}	39.4±3.6^{a}	58.3±5^{a}	56.3±4.4^{a}	-121.5±9.4^{d}	-64.2±31.2^{bc}	20.6±2.9^{abc}	-59.4±4.8^{bc}
**Fo‡‡**	33.7±24.9^{abc}	47.1±10.3^{abc}	22.2±16.7^{abc}	34.7±23^{abc}	-86.5±47.4^{cde}	-68.7±11.5^{bcde}	-16.2±27.6^{abcde}	-5.5±6.9^{abcd}
**Pe§§**	32.2±26.9^{abcd}	17.7±13.1^{abcd}	33.4±17.9^{abcd}	40.6±11.1^{abc}	-116±47.5^{cde}	-57.9±21.6^{bcde}	-22.2±32.2^{abcde}	-119.2±15^{cde}

Classification of Antimicrobial activity: > 50 % = strong inhibition; 15- 50 % = moderate inhibition; <14.99 % = No inhibition.

Culture media: FOB: Forage oats broth, CWB: Cheese whey broth, FFB: Flour fish broth.

Indicator microorganisms: **Bc*: *B. cereus*, †*Lm*: *L. monocytogenes*, ‡*Sa*: *S. aureus*, §*Ca*: C. *albicans*, ¶*Sc*: *S. cerevisiae*, ***Ct*: *C. tropicalis*, ††*n*: *A. niger*, ‡‡*Fo*: *F. oxysporum*, §§*Pe*: *P. expansum*.

Different letters correspond to significant differences on each row, for each growth media analyzed $P < 0.05$.
